# Vehicle image datasets for image classification

**DOI:** 10.1016/j.dib.2024.110133

**Published:** 2024-02-01

**Authors:** Narong Boonsirisumpun, Emmanuel Okafor, Olarik Surinta

**Affiliations:** a Department of Computer Science, Loei Rajabhat University, Loei 42000, Thailand; b SDAIA-KFUPM Joint Research Center for Artificial Intelligence, King Fahd University of Petroleum and Minerals, Dhahran 31261, Saudi Arabia; c Multi-agent Intelligent Simulation, Laboratory (MISL) Research Unit, Department of Information Technology, Faculty of Informatics, Mahasarakham University, Mahasarakham, 44150 Thailand

**Keywords:** Vehicle type image, Vehicle make image, Vehicle logo, Thai vehicle image, Vehicle image recognition, Image classification

## Abstract

Vehicle image recognition is a critical research area with diverse traffic management, surveillance, and autonomous driving systems applications. Accurately classifying and identifying vehicles from images play a crucial role in these domains. This work presents two vehicle image datasets: the vehicle type image dataset version 2 (VTID2) and the vehicle make image dataset (VMID). The VTID2 Dataset comprises 4,356 images of Thailand's five most used vehicle types, which enhances diversity and reduces the risk of overfitting problems. This expanded dataset offers a more extensive and varied collection for robust model training and evaluation. This dataset will be valuable for researchers focusing on vehicle image recognition tasks. With an emphasis on sedans, hatchbacks, pick-ups, SUVs, and other vehicles, the dataset allows for developing and evaluating algorithms that accurately classify different types of vehicles. The VMID Dataset contains 2,072 images of logos (called vehicle make) from eleven prominent vehicle brands in Thailand. The proposed dataset will facilitate the development of computer vision algorithms and the evaluation of learning algorithm model performance metrics. These two datasets provide valuable resources to the research community that will foster possible research advancements in vehicle recognition, vehicle logo detection or localization, and vehicle segmentation, contributing to the development of intelligent transportation systems.

Specifications Table

**Table:** 

Subject	Computer Vision, Computer Science
Specific subject area	Computer Vision, Image Processing, and Image Classification
Data format	Raw
Type of data	Image
Data collection	The vehicle image datasets were collected through CCTV cameras installed at the front gate of the Loei Rajabhat University in Thailand. We captured all vehicles passing through the university gate using CCTV cameras in various natural environments, capturing changing weather conditions, lighting, focus, occlusion, resolution, distance, and angle. Furthermore, we categorized the vehicle images into two distinct datasets: the vehicle type image dataset version 2 (VTID2) and the vehicle make image dataset (VMID).
Data source location	Video surveillance system of Loei Rajabhat University (Main Gate).
Data accessibility	Repository name: Mendeley Data Vehicle Type Image Dataset (Version 2) – VTID2: Data identification number: 10.17632/htsngg9tpc.3 Direct URL to data: https://data.mendeley.com/datasets/htsngg9tpc/3 Vehicle Make Image Dataset (VMID): Data identification number: 10.17632/8ssr6kptbx.2 Direct URL to data: https://data.mendeley.com/datasets/8ssr6kptbx
Related research article	Boonsirisumpun, N., & Surinta, O. (2022). Ensemble multiple CNNs methods with partial training set for vehicle image classification. *Science, Engineering and Health Studies*, 16, 22020001. DOI: 10.14456/sehs.2022.12

## 1 Value of the Data

• The datasets provide valuable resources for researchers working on vehicle image recognition, offering a diverse collection of vehicle images for accurate classification and identification tasks.
• Researchers in computer vision, artificial intelligence, and transportation can benefit from these datasets to develop and evaluate algorithms, models, and techniques related to vehicle image recognition.
• The datasets will provide an avenue for the scientific community to evaluate the performance of their learning algorithms and models of vehicle image recognition, hence contributing to research advancements.

## 2 Objective

These datasets were generated to advance the field of vehicle image recognition. The rationale behind their creation was driven by the need to provide researchers with a comprehensive and diverse collection of vehicle images for accurate classification and identification tasks. The datasets capture the most used vehicle types in Thailand, ensuring relevance to the local context, and were expanded to mitigate overfitting risks. Additionally, vehicle make recognition emphasizes the importance of logo analysis in classifying vehicles based on their unique design characteristics. Overall, these datasets serve as valuable resources for researchers to develop and evaluate algorithms, models, and techniques, ultimately contributing to vehicle image recognition research advancement.

## 3 Data Description

**Fig. 1:**
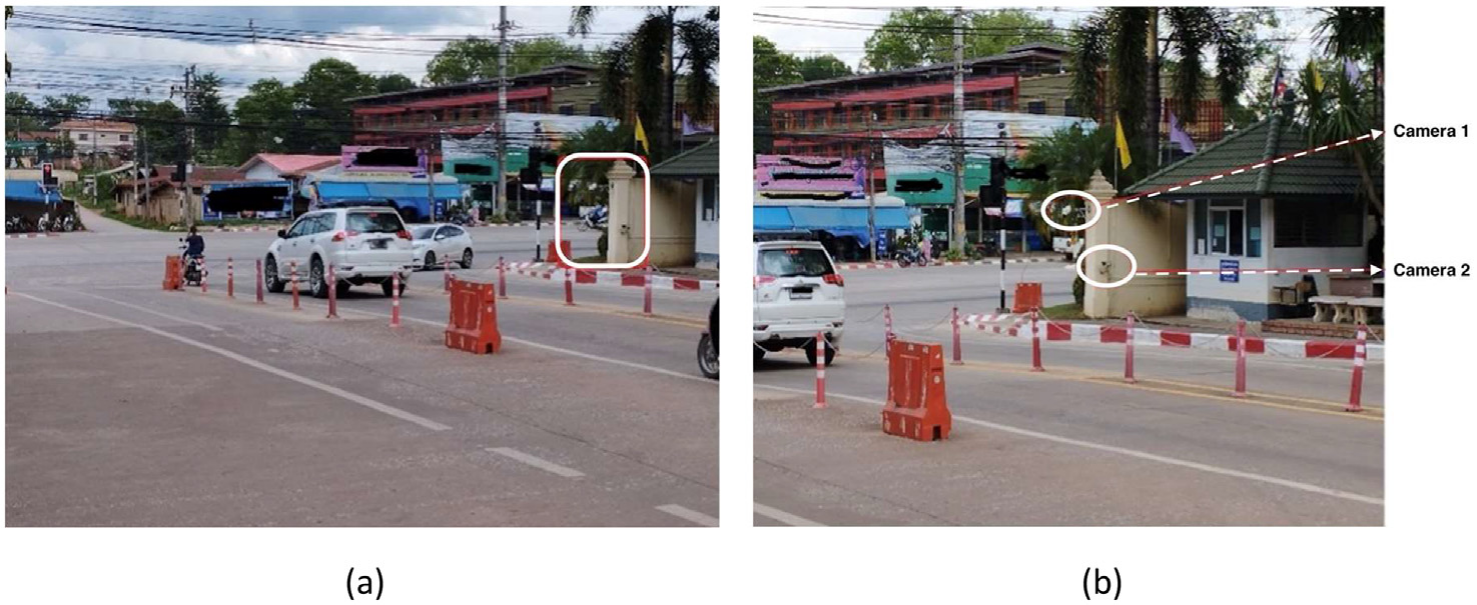
The location of the installed CCTV cameras at the front gate of the university. (a) Image of the front gate area and (b) Positions of cameras 1 and 2.

The vehicle type image dataset aims to investigate five prevalent vehicle types used in Thailand, namely sedans, hatchbacks, pick-ups, SUVs, and vans. The images were collected using recording devices from a video surveillance system at Loei Rajabhat University in Loei province, Thailand. The data was collected over four months from July to December 2018 using two cameras installed at the front gate of the university, as shown in Fig. 1.

**Fig. 2:**
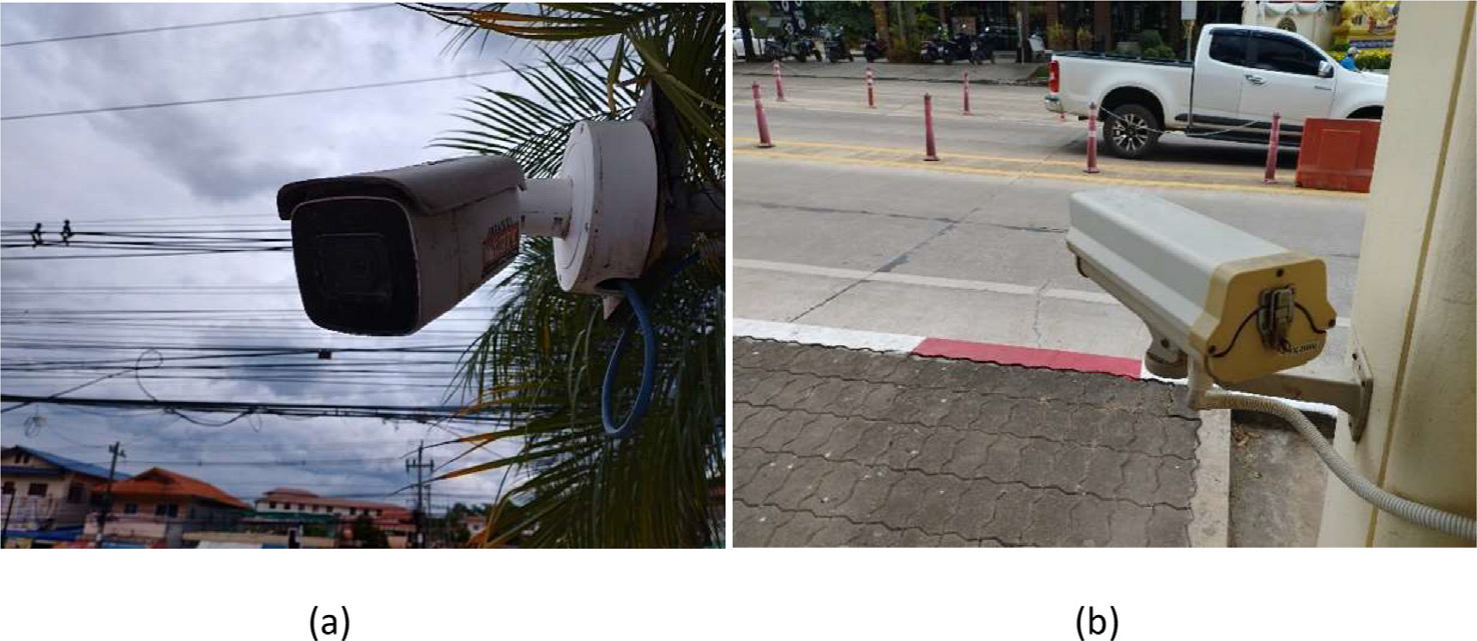
The position of two installed CCTV cameras at the front gate of the university: (a) Camera 1 and (b) Camera 2.

The positions of both cameras are illustrated in Fig. 1. As shown in Fig. 1(b), The first camera is installed at the top position of the pole but angled downward at a 45° angle, capturing an overhead perspective. The second camera is mounted at the bottom position that projects an inclined visual-view from a lower perspective (Fig. 2).

However, to address the relatively limited number of van images compared to other types of vehicles, additional vehicle-type images, including motorcycles, were included within the van category. Consequently, the van category was renamed “Other vehicles” to augment dataset diversity. The vehicle type image dataset (version 1) (VTID1) [1] includes 1310 sample images: 400 sedans, 478 pick-ups, 129 SUVs, 181 hatchbacks, and 122 images of other vehicles. Each image was captured from a video with a resolution of 1280 × 720 pixels and subsequently extracted to isolate the vehicle image using the single-shot multi-box detector (SSD) model [2]. Given its substantial size, comprising over a thousand images and more than a million parameters, the dataset is particularly suitable for training deep learning models. The researcher can download the VTID1 dataset to preliminary test new deep learning algorithms from the following URL: https://data.mendeley.com/datasets/r7bthvstxw/2

**Fig. 3:**
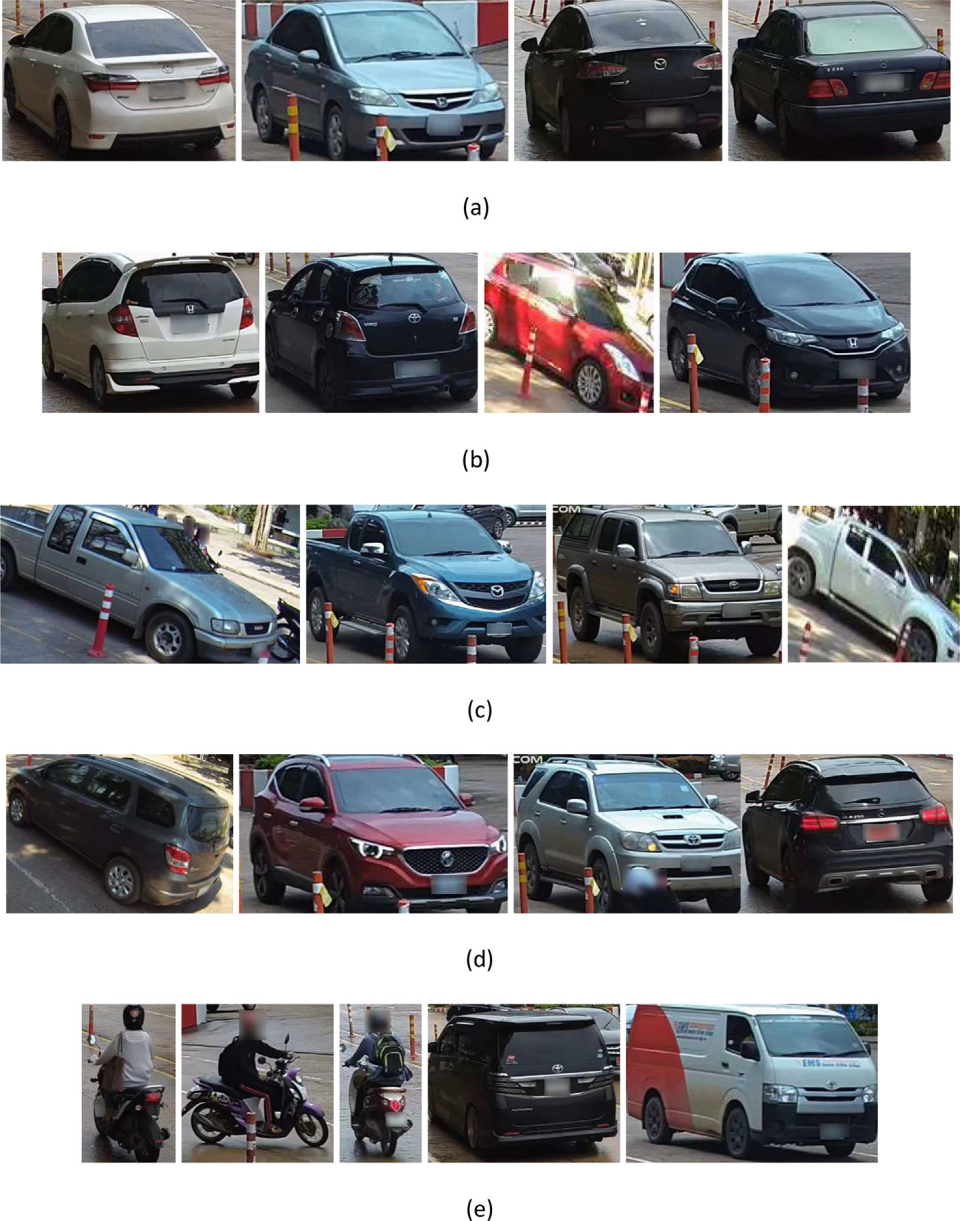
Examples of the VTID dataset: (a) Sedan, (b) Hatchback, (c) Pick-up, (d) SUV, and (e) Other vehicles.

We extended the collection process to augment diversity further, mitigate data overfitting, and generate the vehicle type image dataset (version 2) (VTID2) [3]. The vehicles have been extracted from the original images and stored in various resolutions and JPG format with RGB color space. The vehicle images of the VTID2 dataset have varying sizes, ranging from 5 KB to over 50 KB. The VTID2 dataset contains 4356 images categorized into five categories: 1230 sedans, 1240 pick-ups, 680 SUVs, 606 hatchbacks, and 600 images of other vehicles, as shown in Fig. 3. The increased size and variability of VTID2 provide researchers with an improved resource for developing and evaluating algorithms, models, and techniques in robust vehicle image recognition.

We are concerned about protecting sensitive information like faces and license plates. To achieve this, we made a blur block on the license plates and blurred the faces of individuals in the dataset, as shown in Fig. 3.

**Fig. 4:**
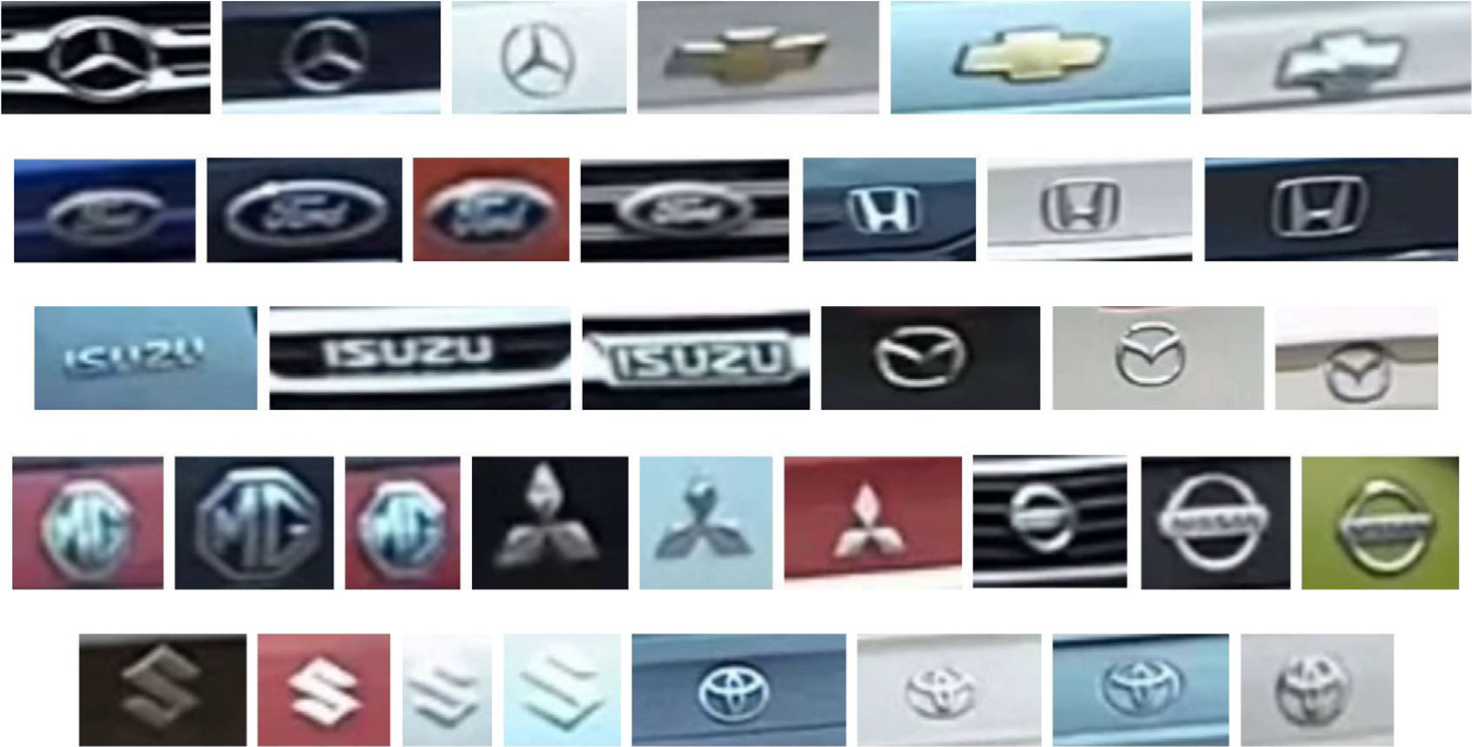
Example images of the VMID dataset.

We also focused on recognizing the make of the vehicles. We then created the vehicle make image dataset (VMID) [4], which includes 2072 images featuring 11 prominent vehicle logos found in Thailand, including Benz, Chevrolet, Ford, Honda, Isuzu, Mazda, MG, Mitsubishi, Nissan, Suzuki, and Toyota, as shown in Fig. 4. The logos of the vehicles are stored in PNG format with RGB color space at various resolutions. The size of the image's ranges from 3 KB to 15 KB.

The VMID dataset facilitates the development and evaluation of algorithms aiming to classify vehicles based on their distinctive logo designs accurately.

## 4 Data Limitation

The limitations considered during the curation of the dataset are described as follows;

1. While collecting the vehicle dataset, we observed human facial contents within the vehicular images in the collected dataset. Hence, it may not be convenient to reveal facial information about individuals without their expressed permission. Therefore, we decided to curb this challenge by blurring the instances of all facial contents in the vehicular images within the dataset and license plate number to avoid conflict of interest. We retained the vehicular bodily texture and logo.
2. While collecting the vehicular images within our dataset, there are no seasonal or climatic considerations besides collecting the data during the daytime.
3. In the context of demographic consideration, we considered the influx and outflux of all vehicular movements through the front gate of the Loei Rajabhat University in Thailand. This information implies that all the vehicular images within our dataset were collected in one specific location.
4. Our dataset is imbalanced, resulting in data bias because the samples of vehicle images per class are not uniformly distributed (The VTID2 dataset contains 4356 images categorized into five classes: 1230 sedans, 1240 pick-ups, 680 SUVs, 606 hatchbacks, and 600 images of other vehicles). Hence, this creates a research opportunity to enable the researcher community to explore the possibility of using diverse data augmentation techniques to operate on vehicular image samples that are deficient within the dataset; this will aid in establishing data balance with the view to creating learning models with good generalization capability.

## 5 Experimental Design, Materials and Methods

The datasets are not divided into training, validation, and test sets. Researchers can choose to partition datasets based on their preferences. For example, Boonsirisumpun & Surinta [5] utilized the initial VTID1 dataset, which was divided into separate sets for training, validation, and testing purposes. The training set consisted of 917 images, while the validation and test sets comprised 131 and 262 images, respectively. The training images were randomly allocated to different folders to facilitate 10-fold cross-validation. All training procedures were conducted from scratch over 20 epochs and subsequently evaluated using five distinct convolutional neural network models (MobileNets [6], VGG16&VGG19 [7], Inception V3 [8], and Inception V4 [9]). The results, as presented in Table 1, indicated that the MobileNets architecture exhibited superior performance compared to the other four CNN architectures in terms of both accuracy and speed.

**Table 1:** The experimental results of CNN models when evaluated on VTID1 dataset according to Boonsirisumpun & Surinta [5].

CNN Models	Accuracy ± S.D.	Training Time (Min.)	Test Time (Min.)
MobileNets	93.40 ± 0.95	18.25	7.29
Inception V4	90.36 ± 1.58	27.53	18.30
Inception V3	88.81 ± 1.80	22.40	10.21
VGG19	79.77 ± 2.04	37.21	29.56
VGG16	77.53 ± 2.22	35.38	27.22

Boonsirisumpun and Surinta (2017) conducted experiments on the VTID2 dataset using 10-fold cross-validation. The CNN models encountered training using nine folds and one fold was utilized for validation purposes. In the experiments, five CNN models, including MobileNet V1, MobileNet V2, Inception V3, Inception V4, and ResNet50, were trained with 20 and 50 epochs and employed to test their accuracy performance. As shown in Table 2, the experimental results demonstrated that MobileNet V2 outperformed other CNN models when training with only 20 epochs. However, when increasing the training epoch, the Inception V3 slightly outperformed the MobileNet V1.

**Table 2:** The experimental results of the CNN models when evaluated on VTID2 dataset according to Boonsirisumpun & Surinta [10].

CNN Models	Accuracy		Training Time (Min.)	
	20 Epochs	50 Epochs	20 Epochs	50 Epochs
MobileNet V1	**94.38**	94.74	50.72	128.32
MobileNet V2	93.56	93.72	**42.18**	**106.54**
Inception V3	91.37	**95.61**	65.87	167.78
Inception V4	92.17	94.38	81.54	208.10
ResNet50	91.24	92.03	74.21	191.23

Another illustrative study involves the utility of the VMID dataset presented by Boonsirisumpun & Surinta [10]. The images within VMID dataset were randomly distributed among various folders to facilitate 10-fold cross-validation, followed by training using two distinct runtime configurations (20 and 50 epochs). Subsequently, the dataset was evaluated using five additional CNN models (MobileNets V1, MobileNet V2 [11], Inception V3, Inception V4, and Resnet50 [12]). The findings, as presented in Table 3, revealed that Inception V3 demonstrated the highest accuracy among the models considered, albeit at the expense of speed when compared to MobileNet V2.

**Table 3:** The experimental results of the CNN models when evaluated on VMID dataset according to Boonsirisumpun & Surinta [10].

CNN Models	Accuracy		Training Time (Min.)	
	20 Epochs	50 Epochs	20 Epochs	50 Epochs
MobileNet V1	90.57	91.83	22.14	63.96
MobileNet V2	89.61	90.17	**18.57**	**52.63**
Inception V3	**90.83**	**92.22**	32.15	84.70
Inception V4	89.82	90.17	43.24	112.56
ResNet50	88.60	89.55	36.52	103.28

## Limitations

Not applicable

## Ethics Statement

The authors have read and follow the ethical requirements for publication in Data in Brief and confirming that the current work does not involve human subjects, animal experiments, or any data collected from social media platforms.

## CRediT Author Statement

**Narong Boonsirisumpun:** Conceptualization, Data Curation, Investigation, Methodology, Resources, Validation, Writing - Original Draft; **Emmanuel Okafor:** Conceptualization, Validation, Writing - Review & Editing; **Olarik Surinta:** Supervision, Conceptualization, Experimental Design, Writing - Review & Editing, Funding Acquisition.

## Data Availability

Vehicle Make Image Dataset (VMID) (Original data) (Mendeley Data) Vehicle Type Image Dataset (Version 2) (Original data) (Mendeley Data)

## Appendix Supplementary materials

Image, application 1
