# Supplementary material for: Vehicle image datasets for image classification
Source: Data Brief. 2024 Feb 1;53:110133. doi: 10.1016/j.dib.2024.110133 (PMC10859268; doi:10.1016/j.dib.2024.110133)
Supplement: Supplementary file 1 [file mmc1.pdf]

Research Article

Science, Engineering and Health Studies
https://li01.tci-thaijo.org/index.php/sehs
ISSN (Online): 2630-0087

Ensemble multiple CNNs methods with partial training set for vehicle image classification

*Narong Boonsirisumpun and Olarik Surinta**

Multi-agent Intelligent Simulation Laboratory (MISL), Department of Information Technology, Faculty of Informatics, Mahasarakham University, Mahasarakham 44150, Thailand

***Corresponding author**: Olarik Surinta olarik.s@msu.ac.th*

***Received**: 4 April 2022*
***Revised**: 23 May 2022*
***Accepted**: 6 June 2022*
***Published**: 20 September 2022*

***Citation**: Boonsirisumpun, N. and Surinta, O. (2022). Ensemble multiple CNNs methods with partial training set for vehicle image classification. Science, Engineering and Health Studies, 16, 22020001.*

ABSTRACT

Convolutional neural networks (CNNs) are now the state-of-the-art method for several types of image recognition. One challenging problem is vehicle image classification. However, applying only a single CNNs model is difficult due to the weakness of each model. This problem can be solved by using the ensemble method. Using the power of multiple CNNs together helps increase the final output accuracy but is very time-consuming. This paper introduced the new ensemble multiple CNNs methods with a partial training set method. This method combined the advantages of the ensemble technique to increase the recognition accuracy and used the idea of a partial training set to decrease the time of the training process. Its performance helped decrease the time taken by more than 60% but it was still able to maintain a high accuracy score of 96.01%, compared to the full ensemble technique. These properties made it a good choice to compete with other single CNNs models.

Keywords: vehicle image classification; ensemble method; multiple CNNs; partial training set

1. INTRODUCTION

Vehicle image classification is an important issue in the world of computer vision. A benefit from understanding information about each vehicle is that it is possible to solve problems in intelligent transport and security systems. For example, controlling the traffic, detecting a specific car, tracking the movement of vehicles, or eventually guiding a self-driving car on the road. This issue can be separated into many problems due to the complex features of vehicle images such as vehicle type, vehicle shape, vehicle color, vehicle model, vehicle make (logo), and vehicle size.

Many algorithms have been chosen to solve these problems. One modern state-of-the-art method is convolution neural networks (CNNs), the complex machine learning model based on a deep neural network. Several successful CNNs models have been introduced since the 2010s. For example, AlexNet (Krizhevsky et al., 2012), VGGNet (Simonyan and Zisserman, 2014), GoogLeNet Inception (Szegedy et al., 2015), ResNet (He et al., 2016), and MobileNets (Howard et al., 2017). Each model had its advantages and effects on many kinds of image recognition problems.

However, even though the performance of CNNs is quite acceptable for image recognition tasks but some problems remain and are described below. The first problem is choosing the best model for the dataset. Selecting only a single CNNs model that runs strongly on each dataset is uncertain. Some datasets probably have more effect on the complex models while others operate properly on simple models. These are challenging. Finding a good match between model and data requires a lot of experiments to be performed. This problem inspired the idea of using multiple models at the same time to help fix the weak point of each model in the prediction. A uniquely effective idea is the ensemble method. This method can

use multiple learning models to run the prediction separately at the same time and then combine the result from a different model or different data to help predict a more accurate and more solid results (Re and Valentini, 2012).

The second problem is the cost of the time taken. CNNs usually require a massive size of training dataset and a very long time to train the model. These problems are tough for the simple CNNs model and even worse for the ensemble method because their use of multiple CNNs together on the full size of the training dataset requires exponential amounts of time in the training process. This research proposed a solution for this issue by using only some parts of the training set, which were called the partial training set, for each CNNs instead of the complete set. This paper intended to show the performance of the ensemble method with multiple CNNs models for vehicle image classification.

2. MATERIALS AND METHODS

2.1 CNNs

CNNs are the modern algorithm for image recognition that found success, starting in the 2010s. For example, AlexNet, VGGNet, GoogLeNet or Inception, ResNet, and MobileNets. Each model had a different structure, size, and performance on the image classification problem. This research chose five recently state-of-the-art models to perform the experiment with the ensemble method.

2.1.1 MobileNets V1 and V2

MobileNets is a tiny CNN model (4.2 M parameters) using the concept of depthwise and pointwise separable convolution to reduce the model size to be suitable for a mobile platform (Howard et al., 2017). The second-generation (MobileNets V2) followed in 2018 (Sandler et al., 2018) and was somewhat smaller than the previous one (3.4 M parameters). Both models were good in terms of accuracy and speed.

2.1.2 GoogLeNet inception V3 and V4

GoogLeNet or Inception was created by Christian Szegedy in 2014. The recently stable version was V3 and V4 (Szegedy et al., 2016 and 2017). This model could be considered as a medium-size CNNs method with 24 M parameters, which was a lot smaller, compared to AlexNet, but with higher performance. GoogLeNet was one of the standard models used in image recognition tasks (Szegedy et al., 2015).

2.1.3 ResNet50

ResNet was introduced by Kaiming He in 2016, choosing the residual learning block as its core structure (He et al., 2016). This paper chose this model for the experiment as another medium-size CNNs example.

These three methods found success in several vehicle image recognition approaches. For example, Špaňhel applied MobileNets and Resnet50 in vehicle type and color recognition, which improved the accuracy of low-power devices (Špaňhel et al., 2018). Puarungroj studied the performance of Inception-V3 and performed experiments on vehicle license plate images (Puarungroj and Boonsirisumpun, 2018). Thomas used the combination of Inception and Resnet model for the recognition on moving vehicle (Thomas et al., 2020), while Goh implemented the transfer learning MobileNets and achieved higher accuracy and low latency on a real-time vehicle dataset from a video surveillance system (Goh, 2021).

2.2 Ensemble multiple CNNs methods

Ensemble methods are learning algorithms that combine a set of model classifiers and then take the vote or weight summary of their predictions to make a final answer (Polikar, 2012). The original technique used was Bayesian averaging (Dietterich, 2000). Ensemble methods can be used to connect different kinds of model predictors, such as binary tree, support vector machine, and neural network. Each model predicted its own result and then used several ways to combine their prediction. The examples of the combination technique are techniques such as majority voting (Raza, 2019), weight average (Dogan and Birant, 2019) and unweighted average (Sewell, 2011). This research proposed using the two simplest ensemble techniques, the unweighted average method and the majority vote method, with several CNNs classifiers.

2.2.1 Unweighted average method

The unweighted average method is the ensemble method that computes the final prediction of multiple CNNs models by summarizing the probabilities of all models and then dividing that by the number of the models (averaged probability). This method gave the final prediction from the highest probability answer as a result, which can be defined by equation (1):

$$\hat{y}_i = \frac{1}{n}\sum_{i=1}^{n} y_i \tag{1}$$

where y_i is the output probabilities of each CNNs model and n is the number of the models.

2.2.2 Majority vote method

The majority vote method is the simple ensemble method that computes the final prediction of multiple CNNs models by directly counting the result of each model using the argmax function. Then the maximum vote was decided which can be defined by equation (2):

$$\hat{y}_i = \frac{1}{n}\sum_{i=1}^{n} argmax(y_i) \tag{2}$$

where y_i is the output probabilities of each CNNs model and n is the number of the models.

2.3 Ensemble multiple CNNs methods with partial training set

Ensemble multiple CNNs methods were effective ways to summarize the prediction from many CNNs classifiers. They gave higher accuracy of prediction but unfortunately, were compromised by having much longer training times. Because of using more than one classifier, each CNNs needed to be trained separately with a full-size training dataset.

2.4 Dataset

These experiments used two types of vehicle image datasets to perform experiments, revealing the effect of the proposed method on vehicle image classification. The first

one was the vehicle type image dataset (VTID) and the second was the vehicle make image dataset (VMID). Both datasets have been collected from the video surveillance system of Loei Rajabhat University in Loei province, Thailand.

2.4.1 Vehicle type

A vehicle type is the description of the vehicle category that helps define the terms for classifying cars or other types of vehicles. In this research, we focused on five types of mainly personal vehicles in Thailand (Figure 1).

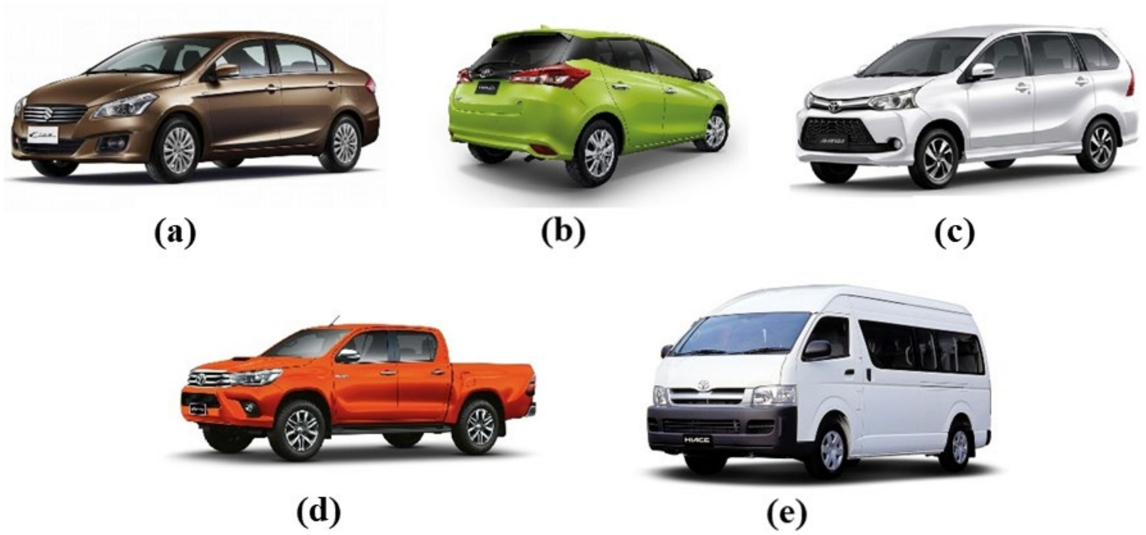

Figure 1. Five types of mainly personal vehicle in Thailand, (a) sedan, (b) hatchback, (c) sport utility vehicle (SUV), (d) pick-up, and (e) van

Note: Sedan: a passenger car with body of two or four doors and two full-width seats inside
Hatchback: another passenger car which has a single rear door for storage
SUV: a larger hatchback that is similar to a station wagon
Pick-up: a small truck with an open body and enclosed cab
Van: a vehicle with three or four passenger seats that can transport more than ten people

2.4.2 Vehicle make

The vehicle's make is the brand of the vehicle and mostly the name of the company manufacturing the vehicle. People easily recognize the vehicle by seeing the logo because of its unique design and is familiar to most people (Figure 2). This can help a machine do the same thing. By locating and recognizing the vehicle logo, it is possible for a computer system to classify the vehicle make by analyzing the differences in each logo and figuring out how to categorize them.

The first dataset, VTID was a collection of five types of popular vehicles as described above. There were two versions of this dataset, VTID1 and VTID2. VTID1 was the smaller set consisting of 1,310 sample images. VTID2 was larger with a total of 4,356 images (Boonsirisumpun and Surinta, 2022). This paper used only VTID2 for the experiment (Figure 3a).

The second dataset, VMID, was the collection of eleven vehicle logos in Thailand (Benz, Chevrolet, Ford, Honda, Isuzu, Mazda, MG, Mitsubishi, Nissan, Suzuki, and Toyota). The total number of images was 2,072 (Figure 3b).

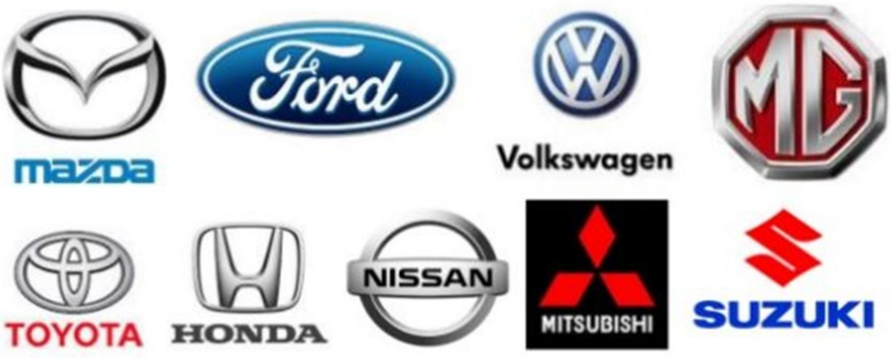

Figure 2. Example of the vehicle logo in Thailand

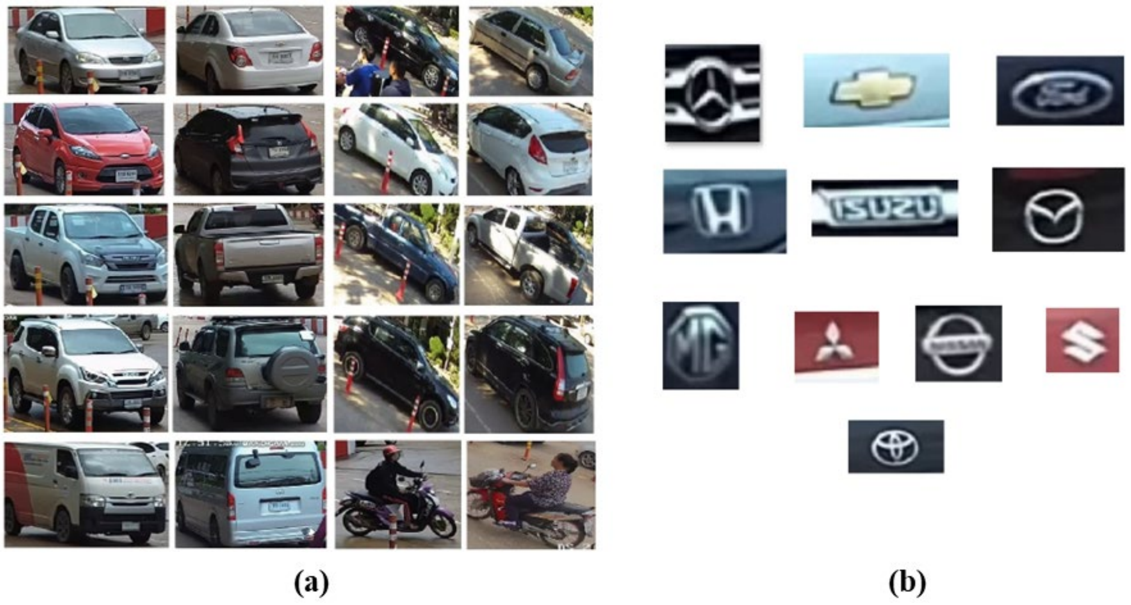

Figure 3. Example of (a) VTID2 and (b) VMID datasets

2.5 Experimentals

The study of the effect of ensemble multiple CNNs methods with a partial training set for vehicle image classification in this research was separated into three steps. The first experiment was the performance of a single CNNs model on the full VTID2 and VMID datasets. The second was the ensemble of five CNNs models on the full VTID2 and VMID datasets. The last one was the ensemble of five CNNs models on partial VTID2 and VMID datasets.

2.5.1 Single CNNs model on full training set

The first experiment was designed to collect the initial performance of each CNNs on the dataset. By using every single CNNs model from the five chosen models (MobileNets V1, MobileNets V2, Inception V3, Inception V4, ResNet50) on the full-size training dataset with no ensemble technique. A 10-fold cross-validation was used to average the accuracy of the preprocessing. The experiments were run using Python 3.7.3 on an Intel Core i-7, 8th Gen, 4.0 GHz, Ram 8GB. The training method used the train from scratch for 50 epochs to compare with last year's experiment (20 epochs). (Figure 4).

2.5.2 Ensemble multiple CNNs model on full training set

The second experiment was designed to study the effect of the ensemble technique on vehicle image classification. By using the same 10-fold cross-validation training dataset from the previous experiment, it replaced the single CNNs classifier using the ensemble of five CNNs models prediction together (MobileNets V1, MobileNets V2, Inception V3, Inception V4, and ResNet50). The combination of the output used both techniques from the ensemble method described in section 2, the unweighted average and majority vote. The experimental results recorded both the accuracy and time consumption to compare with the other experiments (Figure 5).

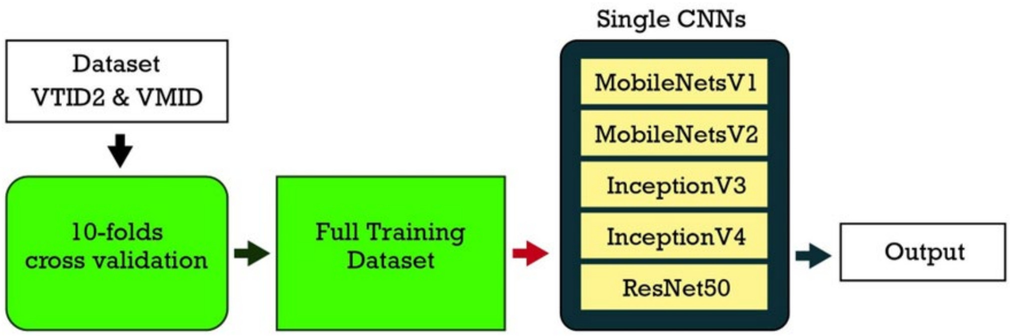

Figure 4. Processes of the first experiment

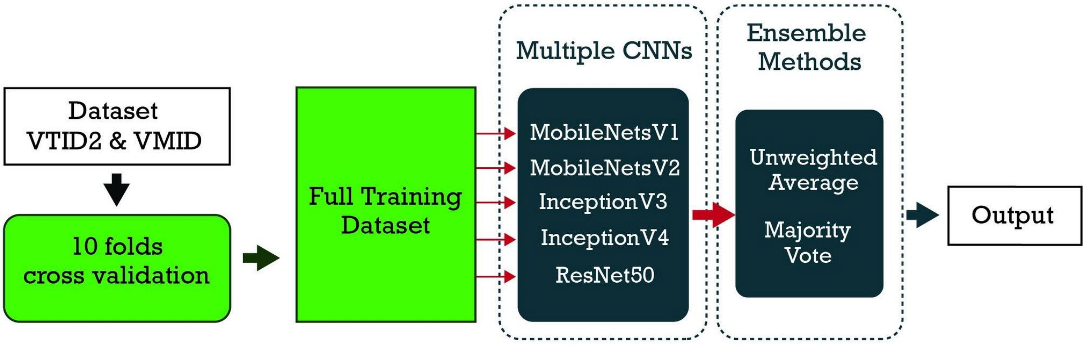

Figure 5. Processes of the second experiment

2.5.3 Ensemble multiple CNNs model on partial training set

The final experiment was designed to study the effect of the partial training dataset on the ensemble methods. By randomly selecting some sliced parts of the training set for each CNNs model. The size of the partial training set was chosen from three parameters (1/2, 1/3, and 1/5 of the full size). Five CNNs models from the previous experiments were also used. The unweighted average and majority vote were still performed. The experimental results recorded both the accuracy and time consumption as in the last experiment (Figure 6).

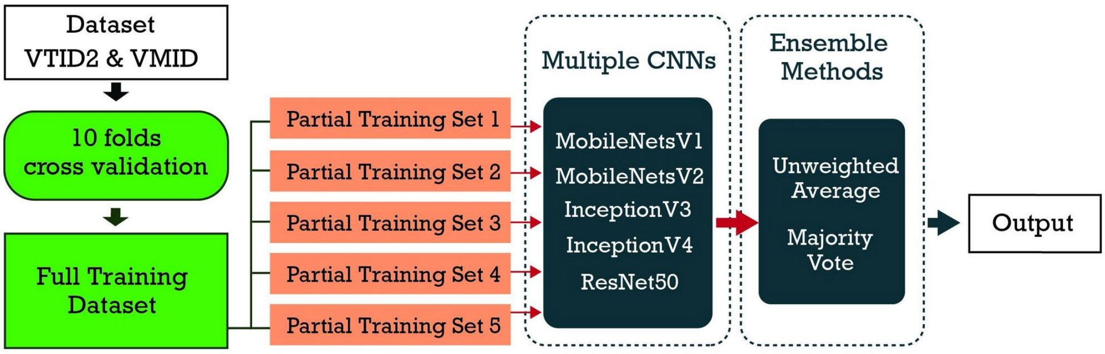

Figure 6. Processes of the third experiment

3. RESULTS AND DISCUSSION

The results of the first experiment are shown in Table 1. These experiments were run using five CNNs models with two different epochs (20 and 50). The result showed that in 20 epochs, the performance of MobileNets V1 was close to that of Inception V3 with MobileNets V1 having the highest accuracy on VTID2 but Inception V3 was better on VMID. However, in 50 epochs, the inception V3 was overcome on both datasets. It could be concluded that the smaller model like MobileNets learned faster than the other when using fewer epochs. But with more epochs, the larger model could find the output better.

The training runtime of the first experiment is shown in Table 2. MobileNets V2 showed the advantages of the smallest model both in two datasets and a different number of epochs (20 and 50). The best result was on the VMID dataset, with only 18.57 minutes to finish the training process in 20 epochs. For VTID2, it seemed like the runtime was double, like the size of VTID2, which is twice the size of VMID. It could probably be concluded that the training time was increased following the ratio of increasing data and epochs.

The results of the second experiment are shown in Table 3. These experiments were challenged by the ensemble of five CNNs models in three different ways (ensemble of five MobileNet V1, ensemble of five Inception V3, and ensemble of the different five CNNs) using 50 epochs both in the unweighted average method and majority vote method. The results showed that the ensemble of the different five CNNs had the highest accuracy both in VTID2 and VMID, in which the unweighted was better than the majority vote. It could be concluded that the combination of different models could help to cover the weaknesses of other models better than using the same model five times.

The training runtime of the second experiment is shown in Table 4. The ensemble of five MobileNets V1 showed the fastest training speed in both of the two datasets. The five models in combination were slightly faster than five Inception V3 but, unfortunately, all three ensembles were much slower than the single CNNs methods. It could be concluded that the good accuracy of the ensemble method required a very long time in the training process.

Table 1. The accuracy of single CNNs method

Network model/dataset	VTID2 (20 epochs)	VTID2 (50 epochs)	VMID (20 epochs)	VMID (50 epochs)
MobileNets V1	94.38	94.74	90.57	91.83
MobileNet V2	93.56	93.72	89.61	90.17
Inception V3	91.37	95.61	90.83	92.22
Inception V4	92.17	94.38	89.82	90.17
ResNet50	91.24	92.03	88.60	89.55

Table 2. The training runtime (min) of single CNNs method

Network model/training time	VTID2 (20 epochs)	VTID2 (50 epochs)	VMID (20 epochs)	VMID (50 epochs)
MobileNets V1	50.72	128.32	22.14	63.96
MobileNet V2	42.18	106.54	18.57	52.63
Inception V3	65.87	167.78	32.15	84.70
Inception V4	81.54	208.10	43.24	112.56
ResNet50	74.21	191.23	36.52	103.28

Table 3. The accuracy of ensemble multiple CNNs method on full training set

Ensemble multiple model/ dataset	Full VTID2 (50 epochs)		Full VMID (50 epochs)	
	Unweighted average	Majority vote	Unweighted average	Majority vote
5 MobileNets V1	95.33	94.98	92.37	91.83
5 Inception V3	96.01	95.93	92.89	92.54
5 models combination	96.15	95.89	93.11	92.89

Table 4. The training runtime (min) of ensemble multiple CNNs method on full training set

Ensemble multiple model/training time	Full VTID2 (50 epochs)	Full VMID (50 epochs)
5 MobileNets V1	650.15	320.75
5 Inception V3	864.76	433.25
5 models combination	803.13	420.56

The results of the third experiment are shown in Table 5. These experiments were focused on the effect of the partial training set technique on the ensemble method. The results showed that when reducing the size of the training set, the accuracy was decreased but was still better than the single CNNs. The accuracy of the 1/2 and 1/3 partial training sets was higher than the highest score on the single CNNs model (Inception V3). It could be concluded that the decreasing of the training set was effect to the accuracy but the power of ensemble different CNNs models helped keep the total accuracy of the combination better than most single CNNs

The training runtime of the third experiment is shown in Table 6. The effect of reducing the training data was obviously less time-consuming. The best size to choose for increasing the speed was a 1/5 partial training set. The overall time could be compared to every single CNNs method and the accuracy of partial training can still be acceptable and higher than single CNNs.

These experiments showed the effectiveness of ensemble multiple CNNs methods compared to a single model on vehicle type and vehicle make image recognition. By using five CNNs models that work together at the same time, the ensemble methods increased the recognition accuracy of both methods but compensated with exponential runtime. To fix the problem of time, the experiment was redesigned using a smaller piece (partial) of the training set instead, and achieved success in solving this time problem while keeping the high accuracy compared to using the full dataset.

Table 5. The accuracy of ensemble multiple CNNs methods on the partial training set

Ensemble multiple model/size of partial training set	Partial VTID2 (50 epochs)		Partial VMID (50 epochs)	
	Unweighted average	Majority vote	Unweighted average	Majority vote
1/2 partial+5 models combination	96.01	95.74	92.94	91.83
1/3 partial+5 models combination	95.85	95.93	92.03	91.65
1/5 partial+5 models combination	95.57	95.01	91.42	90.94

Table 6. The training runtime (min) of ensemble multiple CNNs methods on the full training set

Ensemble multiple model+size of partial training set/ training time	Full VTID2 (50 epochs)	Full VMID (50 epochs)
1/2 partial+5 models combination	402.87	214.88
1/3 partial+5 models combination	276.17	142.67
1/5 partial+5 models combination	172.33	86.74

4. CONCLUSION

In this paper, the researchers proposed a new method, called ensemble multiple CNNs methods with partial training set, for vehicle image classification. By using the concept of ensemble method on a multiple CNNs model, and the idea of randomly slicing a small part of the training set to do a partial training instead of full training is able to reduce the runtime. The experimental results had satisfying outcomes. The ensembles of five CNNs models help to increase the accuracy of vehicle type and vehicle make (logo) image recognition. The precision of model prediction was improved in every combination and succeeded the previous model using only a single CNNs. The concept of the partial training set was suitable to solve the ensemble runtime problem. Slicing the part of the training set helped to decrease more than 60% of the completed ensemble process, but was also able to keep better accuracy than a single model.

For future work, this new method requires more experiments with other types of problems and a different dataset to ensure its performance. All hyper-parameters need to perform analysis. For example, the number of model combinations can probably be selected from the confidence value of each model instead of a fixed number. Additionally, the size of each partial training set can be weighted based on the performance of every single model instead of choosing the same size. A better fine-tuning parameter will help lead to better performance of the future method.

ACKNOWLEDGMENT

This research project was financially supported by Mahasarakham University.

REFERENCES

Boonsirisumpun, N., and Surinta, O. (2022). Fast and accurate deep learning architecture on vehicle type recognition. *Current Applied Science and Technology*, 22(1), 1-16.

Dietterich, T. G. (2000). Ensemble methods in machine learning. In *Proceedings of International Workshop on Multiple Classifier Systems*, pp. 1-15. Cagliari, Italy.

Dogan, A., and Birant, D. (2019). A weighted majority voting ensemble approach for classification. In *Proceedings of the 4th International Conference on Computer Science and* Engineering, pp. 1-6. Samsun, Turkey.

Goh, Z. L. (2021). Model optimization for vehicle recognition on edge device, Doctoral dissertation, Naval Postgraduate School, USA.

He, K., Zhang, X., Ren, S., and Sun, J. (2016). Deep residual learning for image recognition. In *Proceedings of the IEEE Conference on Computer Vision and Pattern Recognition*, pp. 770-778. Nevada, USA.

Howard, A. G., Zhu, M., Chen, B., Kalenichenko, D., Wang, W., Weyand, T., Andreetto, M., and Adam, H. (2017). MobileNets: efficient convolutional neural networks for mobile vision applications. *arXiv*, 1704.04861.

Krizhevsky, A., Sutskever, I., and Hinton, G. E. (2012). ImageNet classification with deep convolutional neural networks. In *Proceedings of Advances in Neural Information Processing Systems 25*, pp. 1097-1105. Nevada, USA.

Polikar, R. (2012). Ensemble learning. In *Ensemble Machine Learning* (Zhang, C., and Ma, Y., eds.), pp. 1-34. Boston, Massachusetts: Springer.

Puarungroj, W., and Boonsirisumpun, N. (2018). Thai license plate recognition based on deep learning. *Procedia Computer Science*, 135, 214-221.

Raza, K. (2019). Improving the prediction accuracy of heart disease with ensemble learning and majority voting rule. In *U-Healthcare Monitoring Systems* (Nilanjan, D., Ashour, A. S., Fong, S. J., and Borra, S., eds.), pp. 179-196. San Diego, California: Academic Press.

Re, M., and Valentini, G. (2012). Ensemble methods: a review. In *Advances in Machine Learning and Data Mining for Astronomy* (Way, M. J., Scargle, J. D., Ali, K. M., and Srivastava, A. N., eds.), pp. 563-593. New York: CRC Press.

Sandler, M., Howard, A., Zhu, M., Zhmoginov, A., and Chen, L. C. (2018). MobileNetV2: inverted residuals and linear bottlenecks. In *Proceedings of the IEEE Conference on Computer Vision and Pattern Recognition*, pp. 4510-4520. Utah, USA.

Sewell, M. (2011). Ensemble learning. *University College London Research Note*, 11(2), 1-12.

Simonyan, K., and Zisserman, A. (2014). Very deep convolutional networks for large-scale image recognition, *arXiv*, 1409.1556.

Špaňhel, J., Sochor, J., and Makarov, A. (2018). Vehicle fine-grained recognition based on convolutional neural networks for real-world applications. In *Proceedings of 14th Symposium on Neural Networks and Applications*, pp. 1-5. Belgrade, Serbia.

Szegedy, C., Ioffe, S., Vanhoucke, V., and Alemi, A. (2017). Inception-v4, inception-resnet and the impact of residual connections on learning. In *Proceedings of the Thirty-First AAAI Conference on Artificial Intelligence*, pp. 4278-4284. California, USA.

Szegedy, C., Liu, W., Jia, Y., Sermanet, P., Reed, S., Anguelov, D., Erhan, D., Vanhoucke, V., and Rabinovich, A. (2015). Going deeper with convolutions. In *Proceedings of IEEE Conference on Computer Vision and Pattern Recognition*, pp. 1-9. Massachusetts, USA.

Szegedy, C., Vanhoucke, V., Ioffe, S., Shlens, J., and Wojna, Z. (2016). Rethinking the inception architecture for computer vision. In *Proceedings of IEEE Conference on Computer Vision and Pattern Recognition*, pp. 2818-2826. Nevada, USA.

Thomas, A., Harikrishnan, P. M., Palanisamy, P., and Gopi, V. P. (2020). Moving vehicle candidate recognition and classification using inception-resnet-v2. In *Proceedings of IEEE 44th Annual Computers, Software, and Applications Conference*, pp. 467-472. Madrid, Spain.
